# Mechanisms of Interactions between Bile Acids and Plant Compounds—A Review

**DOI:** 10.3390/ijms21186495

**Published:** 2020-09-05

**Authors:** Susanne Naumann, Dirk Haller, Peter Eisner, Ute Schweiggert-Weisz

**Affiliations:** 1 ZIEL-Institute for Food & Health, TUM School of Life Sciences Weihenstephan, Technical University of Munich, 85354 Freising, Germany; dirk.haller@tum.de (D.H.); peter.eisner@ivv.fraunhofer.de (P.E.); 2 Fraunhofer Institute for Process Engineering and Packaging (IVV), 85354 Freising, Germany; ute.weisz@ivv.fraunhofer.de; 3 Chair of Nutrition and Immunology, TUM School of Life Sciences Weihenstephan, Technical University of Munich, 85354 Freising, Germany; 4 Steinbeis-Hochschule, Faculty of Technology and Engineering, George-Bähr-Straße 20, 01069 Dresden, Germany

**Keywords:** dietary fibre, β-glucan, phytochemicals, polyphenol, flavonoid, protein, bile acid binding, bile acid excretion, bile acid profile

## Abstract

Plant compounds are described to interact with bile acids during small intestinal digestion. This review will summarise mechanisms of interaction between bile acids and plant compounds, challenges in in vivo and in vitro analyses, and possible consequences on health. The main mechanisms of interaction assume that increased viscosity during digestion results in reduced micellar mobility of bile acids, or that bile acids and plant compounds are associated or complexed at the molecular level. Increasing viscosity during digestion due to specific dietary fibres is considered a central reason for bile acid retention. Furthermore, hydrophobic interactions are proposed to contribute to bile acid retention in the small intestine. Although frequently hypothesised, no mechanism of permanent binding of bile acids by dietary fibres or indigestible protein fractions has yet been demonstrated. Otherwise, various polyphenolic structures were recently associated with reduced micellar solubility and modification of steroid and bile acid excretion but underlying molecular mechanisms of interaction are not yet fully understood. Therefore, future research activities need to consider the complex composition and cell-wall structures as influenced by processing when investigating bile acid interactions. Furthermore, influences of bile acid interactions on gut microbiota need to be addressed to clarify their role in bile acid metabolism.

## 1. Introduction

Bile acids are a family of molecules that contribute to a variety of key systemic functions in the human body. Bile acids act as detergents facilitating the digestion and absorption of lipids, cholesterol, and fat-soluble vitamins. Recent research activities show that bile acids act as regulators of the gut microbiome and play a key role as signalling molecules by modulating cell proliferation, gene expression, and lipid and glucose metabolism [1,2]. Plant-based food components are considered to interact with bile acids during upper gastrointestinal digestion [3]. By increasing the transfer rates of bile acids from the small intestine into the colon, these interactions may modulate the bile acid pool size and composition, affecting metabolic processes involved in health and disease states. A further understanding of the interactions between bile acids and plant compounds is thus needed in order to recognise related changes of bile acid profiles as a measure of physiological homeostasis [4]. Plausible ways of interactions include interactions at a molecular level as well as bile acids’ retention due to viscous polymer networks [5]. However, the underlying mechanisms, their extent, and their interactions are not yet fully understood.

Investigating interactions of plant compounds with bile acids poses a challenge for research. Due to alterations of bile acids in the human colon and deviating bile acid profiles in many animal models [6], physiological outcomes of in vivo studies provide limited conclusions about the underlying mechanisms. Therefore, a variety of in vitro studies were conducted in the last decades [3]. However, many in vitro studies lack comparability and transferability of the results to physiological processes. For instance, in vivo studies repeatedly indicate a pronounced effect of viscosity and molecular weight of, for example, oat beta-glucan on bile acid excretion [7]. Otherwise, some in vitro studies reported inverse dependencies of increasing bile acid retention capacity for reduced viscosities and molecular weights [8,9]. In a recent comparison of common in vitro methodologies, it was found that these discrepancies between in vitro and in vivo results might be related to an underestimation of viscous effects in certain in vitro assays [10].

To elucidate the cascade of events related to the health attributes of plant-based foods, a considerable number of research activities have addressed the interactions between bile acids and dietary fibre [3]. Dietary fibre may occur in isolated form or as part of complex cell-wall architectures. Therefore, the group of dietary fibres comprises a multitude of different structures [11]. Human ileostomy studies have shown that a diet fortified in dietary fibre from oat induces increased bile excretion within 24 h after consumption [11,12,13]. Most studies thus agree that dietary fibres majorly contribute to bile acid interaction in digests of plant compounds [3,5,11]. However, studies on dietary fibre retaining intact cell-wall structures fail to provide conclusive results regarding the nature and mechanism of the interactions with bile acids [11]. In particular, high variabilities were reported when comparing results on an equal dietary fibre basis for various fruits [14]. Furthermore, a correlation between the dietary fibre composition (proportion of soluble to insoluble fibre) and bile acid sequestering effects could not be established for different fibre preparations derived from fruits, vegetables, or cereals [15]. These studies suggest that bile acid interactions with other plant compounds, such as proteins and phytochemicals, may add to the bile acid retarding effects of dietary fibres [16].

The objective of this review is to provide an update on the most recent findings concerning bile acid interactions with plant compounds. We will first give an overview of proposed interaction mechanisms within the gastrointestinal tract and outline the investigation methods for these interaction mechanisms. We will then explore how these interactions differ as related to bile acid structures and plant tissue compounds (dietary fibre, proteins, and phytochemicals). By this means, we aim to contribute to unravelling the role of bile acid interactions in the health-promoting effects of plant-based foods.

## 2. Bile Acid Metabolism and Chemistry

Primary bile acids, cholic acid (CA) and chenodexoycholic acid (CDCA), are synthesised in the liver by the conversion of cholesterol, which involves 17 distinct enzymes and is accomplished via two different pathways [17]. The first step of synthesis, described to be the rate-limiting step, is catalysed by cholesterol 7α-hydroxylase (CYP7A1). The gene expression encoding CYP7A1 is known to be suppressed by a number of factors, including insulin, protein kinase C activators, cytokines, steroid hormones, and bile acids [6]. The feedback regulation of bile acid synthesis is realised in the liver and the intestine via the farnesoid X receptor (FXR) acting as a bile acid sensor [18].

**Figure 1:**
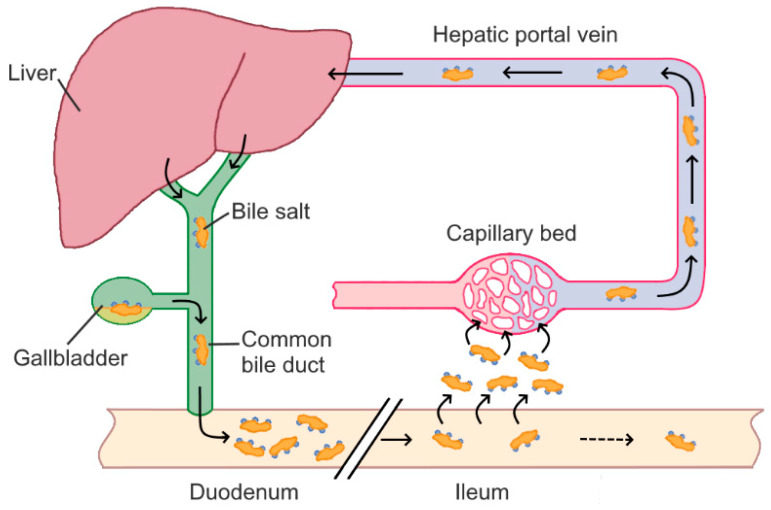
Enterohepatic circulation of bile acids.

The bile acid pool contains about 2.5–5 g of bile acids, which are conjugated either with taurine or glycine to form water-soluble bile salts [19]. Bile salts have different abundancies in bile, with glycoconjugates making up about 70% and tauroconjugates, accounting for 30% of human bile salt mixtures [20]. Bile salts are stored in the gall bladder, which is stimulated to contract and secrete the bile when food passes from the stomach into the duodenum [21]. Bile salts are steroidal detergents, which form mixed micelles with lipids, fats, and/or cholesterol, and thus enable the digestion and absorption of fats and fat-soluble vitamins in the intestine. Conjugated bile acids are reabsorbed mainly by active transport mediated by the apical Na^+^-dependent bile salt transporter, transported back to the liver via the portal circulation, and then re-secreted into the bile. During each cycle of the enterohepatic circulation (Figure 1), about 95% of the bile acids are recovered. The 5% of bile acids lost account for about 400 to 800 mg daily and become substrate to microbial transformation [22].

By the action of anaerobic microbiota, primary bile acids are converted into secondary and tertiary bile acids. The most common secondary bile acids, resulting from deconjugation and dehydroxylation of primary bile acids, are desoxycholic acid (DCA) and lithocholic acid (LCA) [23]. Due to its hydrophobicity, reabsorption rates into the enterohepatic circulation are small for LCA [24]. On the other hand, DCA is reabsorbed in the colon and accumulates in the bile acid pool [17]. The human bile acid pool thus predominantly consists of CA, CDCA, and DCA, accounting for about 40%, 40%, and 20% of the bile acid pool, respectively [6].

**Figure 2:**
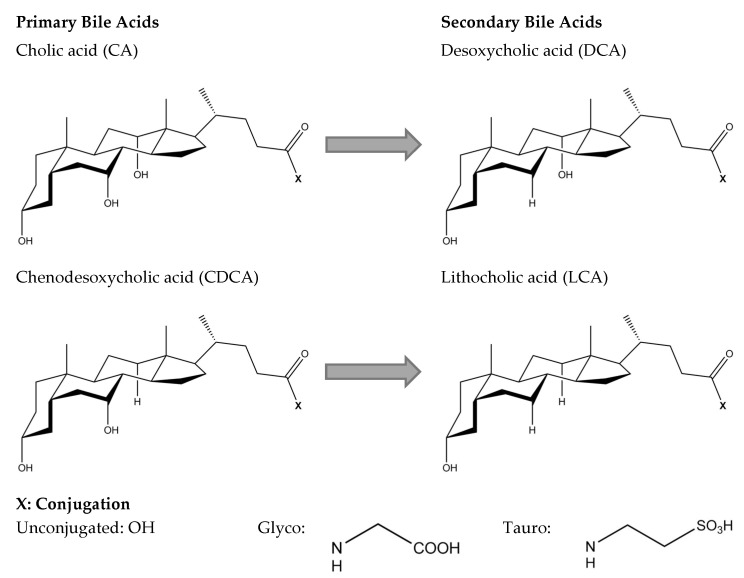
Chemical structure of primary bile acids, cholic acid (CA) and chenodesoxycholic acid (CDCA), and secondary bile acids, desoxycholic acid (DCA) and lithocholic acid (LCA).

The basic chemical structure of all bile acids includes a rigid steroid nucleus and a short aliphatic side chain. Structural differences in the hydroxylation and conjugation of the most common primary and secondary bile acids are given in Figure 2. Bile acids contain hydrophobic and hydrophilic moieties, which makes them facially amphipathic [21]. The amphiphilic character of bile acids is explained by their rigid steroid backbone containing methyl groups oriented towards a hydrophobic face, whereas the hydroxyl groups and the amino group (taurine or glycine) are oriented towards a hydrophilic face [25]. Due to their varying hydroxylation, bile acids differ in hydrophobicity, which decreases in the order of LCA > DCA > CDCA > CA [24].

## 3. Principals and Mechanisms of Bile Acid Interactions

Most studies explaining the interaction of plant compounds and bile acids allow a classification of the interactions into two possible mechanisms, which will be the focus of this review. One of the mechanisms suggests a direct molecular association of bile acids with plant compounds. The other mechanism is based on an increase in the viscosity of the small intestinal content, which leads to a reduced mobility of the bile acid micelles [5,10]. These interactions are proposed to partially prevent bile acids from being reabsorbed into the enterohepatic circulation. Thus, an excess faecal bile acid excretion is considered as an indicator for bile acid interaction in in vivo studies. Accordingly, Ellegard and Andersson [12] reported an increase in bile acid excretion and the activation of CYP7A1 after consumption of oat bran breakfast cereals in ileostomy subjects. Median excretion of bile acids was increased by 144% after a diet including native bran in comparison to a control diet using hydrolysed bran. Therefore, the authors concluded that an increase in bile acid excretion might be caused by the entrapment of bile acid micelles due to the increased viscosity by β-glucan. Similar results were reported for ileostomy bile acid excretion after consumption of highly viscous citrus pectin [26]. Solubility and molecular weight are key factors influencing the viscosity increase of intestinal contents caused by dietary fibres. Therefore, solubility and molecular weight are considered crucial parameters for bile acid interactions, as repeatedly and recently demonstrated for β-glucans [27,28,29]. Nevertheless, some studies fail to establish correlations between the molecular weight and the indicators for bile acid interaction. In particular, a recent study by Iaccarino et al. [28] showed that a diet enriched in structurally different β-glucans increased faecal bile acid excretion in vivo. However, no significant differences were found between a commercial β-glucan preparation with a molecular mass of 100 kDa, and a β-glucan extract with a molecular mass of 530 kDa and a higher viscosity of the resulting feed preparation. On the other hand, Simonsen et al. [30] described molecular interactions between the same commercial β-glucan preparation and bile acids. The in vitro methodology applied in this study excludes the viscous properties of the β-glucan preparation [10]. The results of Simonsen et al. [30] thus indicate that molecular interactions may add to the viscous properties of the commercial β-glucan preparation. The increased bile acid excretion described by Iaccarino et al. [28] could thus be explained by overlapping viscous and molecular effects. Viscous as well as molecular interactions between plant digestive products and bile acids are described to differ depending on the bile acid structures, indicating potential molecular mechanisms [31]. Bile acids mainly abundant in human bile include glyco- and tauro-conjugated CA, CDCA, and DCA. These differ in conjugation and hydroxylation (Figure 2). Differences in viscous and molecular bile acid interactions, as influenced by bile acid structures, will be discussed in Section 3.1 and Section 3.2.

### 3.1. Bile Acid Interactions Related to Viscosity

Increased viscosity during small intestinal digestion is mainly generated by indigested plant polymers, namely dietary fibre. Using an in vitro dialysis model, bile acid diffusion was revealed to decrease in the order CA > CDCA > DCA. For highly viscous samples, like citrus and apple pectin, the bile acid release rate for DCA was reduced by up to 55% compared to CA [31]. In general, diffusion rates decrease with decreasing temperature and increasing viscosity and/or particle radius, as defined by the Stokes–Einstein equation [32]. Stating that the temperature and viscosity during digestion are influenced equally for all bile acids, the diffusion rate depends solely on the radius of the diffusing bile acids. This radius is controlled by the critical micelle concentration (CMC) and aggregation number, which defines the number of monomers within a micelle. These parameters are summarised for the main bile acids abundant in the human bile acid pool in Table 1.

The CMC decreases for CA > CDCA > DCA, while the aggregation number increases for the opposite order. These findings indicate that micellisation depends on the hydrophobic effect, aiming at minimising the hydrophobic surface, and on the hydrogen binding, which is determined by the number, location, and orientation of the hydroxyl groups [33]. Consequently, DCA forms micelles at lower concentrations and includes more monomers compared to CA, which could explain the significant decrease observed in bile acid diffusion for DCA within viscous matrices [31]. Micellar properties of bile acids further depend on solution parameters, such as ionic strength and pH. By increasing the ionic strength, the electrostatic repulsion between micelles is reduced, and aggregation is thus favoured, and CMC decreased. This is especially relevant when comparing results derived for different in vitro conditions. Due to their low pK_α_, conjugated bile acids are almost completely dissociated during digestion. Thus, changes within the physiological pH range do not markedly change the ionisation state and micellisation of bile acids [33].

### 3.2. Bile Acid Interactions on the Molecular Level

Several studies report positive correlation of bile acid sequestering effects with bile acid hydrophobicity, e.g., as described for potato peels, extrudates from barley and oat, or pastry products enriched in buckwheat, chokeberry, and mulberry fractions [35,36,37,38]. However, the exact mechanism of interaction remains to be fully elucidated. In a recent in vitro study, molecular interactions between primary and secondary bile acids and dietary fibre-enriched ingredients from barley, oat, maize, and lupin were revealed [31]. These interactions caused a constant sequestration of bile acids, which was independent of the rheological properties of the preparations. For instance, the investigated oat preparation consisted of high amounts of insoluble fibre (92.6 g/100 g dry matter) and low amounts of soluble dietary fibre (1.4 g/100 g dry matter). Consequently, the viscosity measured after in vitro digestion did not significantly differ in comparison to a blank sample without fibre. Nevertheless, the oat preparation significantly adsorbed bile acids (up to 2.6 µmol/100 g dry matter). These results are consistent with a study investigating a different oat preparation after thermal treatment [39]. Due to the thermal processing, the viscosity measured under simulated physiological conditions was almost completely lost. However, the authors reported a dose-dependent bile acid sequestering effect, which reached up to 26% of the bile acid adsorbing effect of the anionic agent cholestyramine. Similar results were reported for oat and barley extracts and hydrolysates with low molecular weight and viscosity [40,41,42].

The referenced studies repeatedly report a constant pattern of molecular interaction, showing increased interaction of dihydroxy bile acids (CDCA and DCA) compared to trihydroxy bile acids (CA). These results indicate that hydrophobic interactions are core to the molecular interactions with plant compounds. Potential contributors to these interactions will be discussed in further detail, while focusing on different plant tissues, in Section 5.

## 4. Analysis of Bile Acid Interactions

The suitability and limitations of in vivo and in vitro studies for the detection and differentiation of viscous and molecular bile acid interactions are displayed in Table 2 and will be discussed in the following Section 4.1 and Section 4.2.

### 4.1. In Vivo Approaches to Study Bile Acid Interactions

Due to the minimal bacterial degradation of bile acids, ileostomy studies are very useful in understanding the influence of food components on the ileal bile acid content [5,12]. However, these studies are very limited in use. As bile acids are altered by fermentation and are partially reabsorbed in the colon, excretion of bile acids in more readily available faecal samples can only be correlated to a certain extent with the interruption of the bile acid metabolism [27]. Recent studies further indicate that after an adaption phase to diet interventions, levels of total circulating bile acids are reduced, as demonstrated by β-glucan and arabinoxylan interventions in pig models [43,44]. Thus, although reabsorption rates were decreased and relative faecal excretion rates were increased, an excess of total faecal bile acid excretion was not detected. These concurrent effects on the reduction of circulating bile acids may further hamper conclusions regarding interacting mechanisms. Moreover, the suitability of many animal models is limited for bile acid studies due to deviating bile acid profiles that reduce the transferability to mechanisms in humans [45,46]. For instance, the bile acid pool in mice consists mostly of hydrophilic bile acids, muricholic acids, and cholic acids, and thus differs significantly from the more hydrophobic bile acid pool in humans [6]. Due to these limitations of in vivo studies, only little or no details on the intermediate mechanisms of bile acid interactions can be elucidated from their physiological outcomes [11]. Therefore, in vitro studies mimicking the physiological environment in the small intestine are of major interest in order to elucidate the basic mechanisms and identify hypotheses for targeted in vivo investigation.

### 4.2. In Vitro and Ex Vivo Approaches to Study Bile Acid Interactions

In vitro bile acid binding capacities have been reported for numerous plant-based foods, as recently reviewed by Singh et al. [3]. However, the term bile acid binding is frequently and erroneously used regardless of the underlying mechanisms [10]. Furthermore, the diversity of in vitro model conditions has hampered the ability to compare results across different studies. Most results lack in comparability as methods vary regarding the use and parameters of in vitro digestion as well as the separation approaches to evaluate bile acid sequestering effects. In the intestine, bile acids form mixed micelles with lipids and cholesterol [33,49]. Nevertheless, many in vitro studies apply bile acid concentrations far below CMC (Table 1), which majorly limits transferability to physiological conditions [5]. Centrifugation steps are a common approach to separate unbound bile acids in vitro [3,15,50,51]. However, a recent comparison of in vitro approaches revealed that these approaches may not be appropriate to take into account the effects of viscous digestive matrices [10]. Yet, reduction of bile acid diffusion by viscous networks is suggested as a core mechanism of interaction, especially for plant-based foods rich in dietary fibre [43]. On the other hand, diffusion kinetics of a bile acid mixture across a dialysis membrane were demonstrated to include both molecular interactions and viscosity effects [10]. Following this method, a standardised in vitro digestion protocol is applied which includes the addition of bile acid mixtures of typical physiological concentrations [52]. The in vitro digesta are dialysed as a simplified absorption model of the unstirred water layer. First-order diffusion kinetics are analysed and evaluated to differentiate between viscous and permanent molecular interactions [10].

To further study viscosity effects and understand physiological mechanisms, bile acid diffusion can be investigated using ex vivo Ussing chamber experiments. An epithelia membrane separates the Ussing chamber so that each side of the membrane faces a separate chamber half, which is filled with physiological solutions [53]. In a study by Gunness et al. [43], tissues from proximal jejunum, mid-jejunum, and terminal ileum derived from pigs were used to study diffusion kinetics. After the addition of oat β-glucan to the mucosal side, a significant decrease in the uptake of a model bile acid across the terminal ileum was reported. Further ex vivo studies focus on changes of intestinal mucus after specified diets to elucidate influences on mucosal permeability, which may add to bile acid sequestering effects, especially for soluble fibres [54].

To elucidate the nature of molecular interactions, a variety of structural techniques can be used. These include nuclear magnetic resonance (NMR) methods to study chemical shift changes in bile salt resonances as a function of the concentration of digested plant compounds [55,56,57]. Additionally, small-angle X-ray and/or neutron scattering, microcalorimetry, surface plasmon resonance analysis, and molecular docking experiments can be performed to elucidate binding stoichiometry and energetics of interactions [58,59].

## 5. Bile Acid Interactions as Related to Plant Tissues

### 5.1. Interactions between Bile Acids and Dietary Fibres

Dietary fibres are classified into soluble and insoluble fibres according to their solubility in water, as it is associated with different physiochemical and functional properties [60]. Soluble dietary fibres are attributed a high water binding capacity, viscosity increasing capacity, and fermentability by intestinal microbiota [5,61,62]. On the other hand, insoluble dietary fibres are described as less fermentable and only have little effect on viscosity in the gastrointestinal tract [63,64]. In 1967, Cookson et al. [65] first published on the relationship between dietary fibre and bile acids focusing on the modification or prevention of cholesterol-induced atherosclerosis. Since then, numerous studies have focused on the interaction between soluble and insoluble dietary fibre and bile acids, resulting in several hypotheses about potential interaction mechanisms [3,11].

The interaction of most soluble dietary fibres, such as pectin, β-glucan, and gums, is mostly ascribed to their viscous properties [5]. Accordingly, Gunness et al. [50] investigated in vitro bile acid diffusion in the presence of wheat arabinoxylan and barley mixed linkage β-glucan and found that the viscous polymers slowed down the passage of bile acid micelles. A permanent molecular interaction between the two soluble fibres and bile acids was not found. However, non-permanent molecular interactions depended on the source of the fibre, and was revealed by NMR and small-angle X-ray scattering analyses [59]. These interactions were classified in two main classes. β-glucan caused mostly small chemical shift changes in the NMR bile acid resonances, indicating dynamic interactions leading to effective local changes of micellar bile concentrations. On the other hand, arabinoxylan mainly reduced the intensities of NMR bile acid resonances, indicating trapping of bile micelles within polymer aggregates. A recent study further focused on the oxidation and partial hydrolysis of β-glucan and confirmed that the bile acid sequestering effect of β-glucan can primarily be ascribed to its viscous properties [29]. Accordingly, the in vitro study revealed that the most viscous native β-glucan extracts exhibited the strongest retardation of bile acid diffusion. In agreement with these findings, reduced reabsorption of bile acids was found after short-term in vivo interventions with β-glucan and arabinoxylan [43,44]. In vivo interventions with structurally different guar gums or pectins showed increased faecal bile acid concentrations after 3 weeks of exposure [66]. Individual bile acid concentrations varied depending on the degree of pectin methoxylation and molecular weight of guar gums and were accompanied with changes in bile acid distribution compared to a fibre-free diet. The correlation between bile acid interaction and pectin structures and viscosity was further shown in vitro, and showed an increased interaction depending on the bile acid hydrophobicity [67,68]. An early study by Pfeffer et al. [69] focused on pectin bile acid interactions using NMR and dialysis experiments. The authors could not find permanent molecular interactions for a purified fraction of native high-molecular pectin but revealed bile acid adsorbing properties for contaminants in commercial pectin preparations. On the other hand, molecular docking experiments performed by Singh et al. [3] suggested binding affinities between pectin and bile acids, which decreased for GCA > GDCA > CA > DCA > CDCA > GCDCA. Contrarily, Lopez-Pena et al. [70] described interactions between sodium taurocholate and pectin based on isothermal titration calorimetry and titration measurements, which suggested that interactions are dominated by hydrophobic forces. To get a deeper insight into molecular mechanisms of pectin bile acid interactions, further studies are needed. From all these studies focusing on structurally different soluble fibres, a central role of viscosity in the interaction with bile acids can be deduced, while no permanent molecular interaction between soluble fibre structures and bile acids is currently established.

In the last decades, interactions of bile acids with samples rich in insoluble fibre were reported for a number of different feedstocks, including barley, oat, rice, wheat, soybean, lupin, and maize [31,71,72]. These interactions were mostly independent of viscosity and increased the dependence on bile acid hydrophobicity. The findings indicated that hydrophobic interactions of insoluble fibres with bile acids could be related to the fibre structures. This hypothesis was investigated/examined in a recent study focusing on cell-wall polysaccarides of lupin cotyledon and hull [73]. Fibres were isolated by proteolytic enzyme treatments followed by alcohol extraction. Purified fibres were sequentially extracted to separate pectin-like, hemicellulosic, and lignocellulosic structures. Bile acid interactions were investigated after in vitro digestion applying dialysis and kinetic analysis. In this study, none of the purified fibre fractions showed a significant molecular interaction with bile acids. Therefore, a major role of lupin cell-wall polysaccharides in molecular bile acid interaction was excluded. The results obtained for cellulose in this study are in line with previous in vitro results focusing on commercial cellulose preparations, which repeatedly conclude that cellulose provides low bile acid binding capacity [10,14,15,51]. Otherwise, Singh et al. have recently modelled the interactions of several BA (unconjugated CA, DCA, CDCA, and their glycoconjugated counterparts) with cellulose through molecular docking, and provided the estimated strength of the binding energy [3]. They showed that GDCA, CA, and GCA had larger binding affinity than DCA, CDCA, and GCDCA. Further studies are thus needed to elucidate potential binding mechanisms. The molecular interactions described for cellulose esters vary greatly from crystalline cellulose preparations and were recently reviewed by Macierzanka et al. [11]. Although no direct molecular interaction between insoluble dietary fibres and bile acids has been established so far, in vivo studies nevertheless indicate interfering activities of insoluble dietary fibres on bile acid metabolism. Accordingly, van Bennekum et al. [63] described hypocholesterolaemic activity of cellulose in a mice model, which was linked with satiation and satiety effects. In a recent study, the effects of supplemented isoenergetic diets varying in cereal fibre and protein content were investigated in overweight and obese adults. The addition of insoluble cereal fibres was described to significantly increase serum concentrations of most bile acids in obese participants, which was associated with preventive effects on worsening of insulin resistance. On the other hand, bile acid levels remained unchanged in overweight participants. The effects on bile acid metabolic signature were thus related to compensatory increases of the bile acid pool or defective bile acid transport in the insulin-resistant, obese participants [74,75].

Bile acid adsorbing capacity was frequently postulated for lignin [38,76,77,78,79,80]. Lignin has a special position among dietary fibres because it is not a polysaccharide but a phenolic macromolecule [81]. Hydrophobic interactions between purified lignin and primary bile acids were revealed in vitro [73]. However, no increase in bile acid adsorption could be achieved by artificial lignification of maize cell walls [82]. Furthermore, no correlation of bile acid adsorption could be established to lignin contents of starchy legumes [83]. It is thus not entirely understood whether lignin adds to molecular interactions observed for dietary fibre-enriched food ingredients [31].

### 5.2. Interactions between Bile Acids and Proteins

Little scientific attention has been paid to the interaction between bile acids and plant proteins [11]. Referring to the bile acid adsorbing capacity of cholestyramine as 100%, high bile acid adsorbing capacities were reported for soy protein (14.5%), pinto beans (5.5%), black beans (8.2%), and wheat gluten (8.8%) by Kahlon and Woodruff [84]. Very high adsorbing capacities were reported for lentil and lupin proteins and hydrolysates, which partly exceeded the values reported for cholestyramine [85,86]. All referenced studies assessed the bile acid adsorbing capacity by centrifugation techniques and measured bile acid concentrations by photometric assay kits. Thus, no details on the molecular mechanisms can be elucidated from the results [11]. Contradictory results were obtained for lupin protein isolates using in vitro dialysis experiments and applying high-performance liquid chromatography for the analysis of individual bile acids. A small but significant bile acid adsorption of CDCA was analysed for a lupin protein isolate, but the adsorption was strongly diminished after alcoholic purification, indicating contributions from associated substances such as flavonoids [16]. Conflicting results were also reported for soybean proteins. Higaki et al. [87] reported that a soybean protein resistant to digestion captures bile acids and stimulates faecal bile acid excretion applying in vitro testing and a rat model. On the other hand, Bosaeus et al. [88] investigated soy bean proteins as alternatives to meat proteins and did not find significant influences on ileostomy bile acid concentrations. A recent study of Wang and Fan [89] focused on structural characteristics of the interaction between zein, a plant protein isolated from maize, and sodium taurocholate. The study showed sodium taurocholate-dependent changes in secondary and tertiary protein structures and electrostatic binding between the bile salt and the protein. However, the investigations were carried out at acidic pH without the addition of digestive enzymes and it is thus unclear to what extent the results can be transferred to physiological conditions in the small intestine. From the current state of the literature, interactions of bile acids with plant proteins are not ruled out, but further studies are needed to specify interactions and potential contributions to health benefits of plant-based foods more precisely. Recent studies further indicate that an increase in the dietary protein content may stimulate bile acid production [74]. These effects on the bile acid metabolism should thus be considered in complement to future interaction studies.

### 5.3. Interactions between Bile Acids and Phytochemicals

Indications of interactions between bile acids and phytochemicals have been repeatedly reported in recent literature, mostly focusing on polyphenolic compounds. In particular, McDougall et al. [90] found substantial alterations in bile acid concentrations of ileal fluids of human ileostomy subjects after consumption of raspberries rich in polyphenols. While unconjugated bile acids were reduced, glyco- and tauro-conjugated cholic acids and desoxycholic acids were increased by up to 120-fold over pre-supplementation levels. Raspberry pomace was further described to favourably alter bile acid profiles in a high-fat mouse model by Fotschki et al. [91].

Also applying a high-fat mouse model, similar results were found for the most abundant green tea polyphenol (−)-epigallocatechin-3-gallate (EGCG) [92]. EGCG supplementation decreased intestinal bile acid concentrations and thus alleviated the potentially harmful expansion of the bile acid pool size typically observed in high-fat diets. Furthermore, EGCG was shown to increase faecal bile acid excretion and significantly increase levels of CYP7A1. Modifications of bile acid and steroid excretion were further reported for a number of polyphenol-rich feedstocks, including apples [93,94], grapes [93,95,96], red beets [93], asparagus roots [97], and peanut skins [98]. Similar physiological outcomes were also described for extracts from black bean seed coats rich in flavonoids and saponins, indicating synergistic effects of these phytochemicals [99]. Saponins, a group of amphiphilic glycosides, have been repeatedly linked to cholesterol-reducing properties in the last decades. Nevertheless, as recently reviewed by Zhao [100], the underlying molecular mechanisms are not elucidated yet.

Based on an NMR study, Ogawa et al. [56] proposed a novel mechanism for the interaction of polyphenols and bile acids. Focusing on tea polyphenols, their results indicate a regiospecific interaction between EGCG or oolongtheanins and bile acids. The authors suggested that these polyphenols form a hydrophobic space that adsorbs the bile acids. Due to this interaction, the micellar solubility of phosphatidylcholine and cholesterol is lowered, thus decreasing absorption and increasing faecal excretion [56]. These results are corroborated by the study of Ikeda et al. [101], who described that polyphenols of black tea decrease cholesterols micellar solubility in vitro and showed that intestinal cholesterol absorption is decreased in rats. Gallic acid, catechin, and epicatechin (0.2 mg/mL) were shown to reduce the micellar solubility of cholesterol by 27%, 12%, and 19%, respectively [102]. Furthermore, Raederstorff et al. [103] reported that the diameter of bile acid micelles was increased when EGCG was added. Applying a model emulsion containing olive oil, phosphatidylcholine, and bile acids, the addition of catechins further markedly increased emulsion droplet size [104]. Li et al. [48] described the formation of a new complex from condensed tannins and bile acids. Interactions were characterised by turbidity, particle size, microstructure, and physicochemical condition analyses. Complementary molecular modelling indicated that the binding occurred through hydrogen bonding and hydrophobic interactions. Furthermore, the stability and digestion properties of bile acid emulsions were analysed, suggesting that the observed complex formation may inhibit lipid digestion and reduce fat absorption.

Bile acid adsorbing capacities of polyphenols were studied applying different in vitro approaches. Yang et al. [105] investigated the bile acid adsorption capacity of kale using an in vitro centrifugation method. The authors found that kale, which is rich in polyphenols and dietary fibre, preferentially bound the hydrophobic bile acids CDCA and DCA. In a second study, the authors compared the adsorbing capacity of raw kale and polyphenol extracts and concluded that certain polyphenolic compounds may have an affinity to adsorb bile acids [106]. Applying a similar method, Hamauzu and Suwannachot [107] further linked the bile acid adsorbing capacity of persimmon fruit to its polyphenols (EGCG, epigallocatechin, epicatechin, and epicatechingallate). Using a dialysis approach, the molecular bile acid interactions of lupins were linked to an alcohol extract rich in flavonoids, showing an increased interaction for CDCA compared to that for CA [16].

The referenced studies indicate that plant polyphenols may interact with bile acids on a molecular level, which may be due to hydrophobic interactions. Besides these direct polyphenol-induced alterations of the bile acid pool, polyphenols are described to significantly change microbiota compositions and affect gen expressions linked to bile acid metabolism [108,109,110]. For instance, resveratrol was reported to decrease ileal bile acid contents, repress FXR, and increase CYP7A1 and bile acid synthesis. However, this functionality was lost after treatment with antibiotics [111]. Therefore, the remodelling of the gut microbiota could represent an alternative physiological mechanism. Future research activities will aim to clarify the role and mechanism of polyphenols in bile acid metabolism.

## 6. Bile Acid Interactions and Influences on Health

Research to investigate the complex interaction between the synthesis of bile acids in the liver, the function of bile acids as signalling molecules, and the intestinal microbiome is at an early stage [2]. It is therefore not possible to draw direct conclusions on the development of diseases and the maintenance of health based solely on interactions between plant components and bile acids. Nevertheless, these interactions may cause an interference with the enterohepatic circulation of bile acids, which plays a core role in nutrient absorption, metabolic regulation, and homeostasis [17]. Plant compounds show variations in interaction with different bile acid species (as discussed in Section 3). Both viscosity-related and molecular interactions have been described to be increased for dihydroxy bile acids (CDCA and DCA) compared to trihydroxy bile acids (CA). Interactions with plant compounds may thus alter the bile acid composition, resulting in a more hydrophilic bile acid pattern. Interactions between bile acids and plant compounds may partially prevent reabsorption of bile acids, which results in changes of the bile acid pool size, an excess excretion, and accumulation of bile acids in the colon. These changes may possibly affect health aspects currently associated with bile acid metabolism.

It is evident that the conversion of cholesterol into bile acids is responsible for the turnover of a major fraction of cholesterol (about 500 mg per day) in humans [17,112]. Thus, bile acid metabolism is directly linked to blood cholesterol levels [113]. Interactions between plant compounds and bile acids may reduce the reabsorption rates of bile acids back into enterohepatic circulation and cause a depletion of bile acids in the liver [5]. Due to the negative feedback regulation of bile acid synthesis, interactions with plant compounds may cause an increase in the conversion of cholesterol to primary bile acids. Accordingly, activations of CYP7A1, the rate-limiting enzyme of bile acid synthesis, were described after diet interventions with plant compounds such as highly viscous apple pectin [114] or tangeretin, a flavonoid derived from citrus peel [115]. These studies indicate that the viscosity-related or molecular interactions described for these plant compounds may contribute to lowering blood cholesterol levels. Plant compounds, especially polyphenols, are further described to change the micellar properties of bile acids. By this means, the micellar solubility of cholesterol and phosphatidylcholin is reduced, and emulsion interface properties are changed [56,104]. Interaction-induced changes in these properties may modify dietary fat digestion and absorption.

Bile acids exert a variety of activities beyond their classical role as fat emulsifiers. Bile acids were identified as endogenous ligands of FXR—a transcriptional regulator of bile acid, glucose, lipid, and energy metabolism. Bile acids are differently potent in activating FXR in the order of CDCA > LCA = DCA > CA [18]. Compositional changes of the bile acid pool may result in a variation in the activation of FXR and consequently affect its regulating function in the metabolism. FXR regulates gene expressions that are involved in the synthesis, uptake, secretion, and intestinal absorption of bile acids, which is reflected in the total bile acid concentrations in the gall bladder [1]. This feedback mechanism depending on bile acid concentrations is important in preventing a potentially harmful expansion of the bile acid pool [116]. FXR further plays a significant role in lipid and glucose homeostasis, as recently extensively reviewed by Shin and Wang [18].

Bile acids research further revealed that bile acids activate the G protein-coupled receptor TGR5, also showing a potency dependence on bile acid hydrophobicity. Amongst others, TGR5 causes the secretion of the gut hormone glucagon-like peptide-1 (GLP-1) [117]. GLP-1 induces the stimulation of insulin secretion and the retardation of gastric emptying, thus contributing to the inhibition of appetite [118]. Interestingly, increased faecal bile acid concentrations by capsulated ileo-colonic delivery of conjugated bile acids were recently shown to increase GLP-1 and improve glucose homeostasis. Thereby, the authors contributed to understanding the effects of bile acids on the human pathophysiology of obesity and diabetes [119].

Recently, emerging research is aiming to clarify the complex interactions between the liver, the bile acids, and the gut microbiome. For instance, the size and composition of the bile acid pool is proposed to add to the regulation of microbial community structures in the gut [2]. Perturbations in the equilibrium between the diet, the gut microbiome, and the bile acid pool size and composition can result in disease states [2]. In particular, high concentrations of secondary bile acids, resulting from microbial transformation of primary bile acids, are reported to promote carcinogenesis in the colon [120,121]. Changes in the bile acid pool are linked to cardiac dysfunctions, liver diseases, biliary stones development, and diabetes. Inflammation, apoptosis, and cell death may be caused by cytotoxicity induced by microbial changes of bile acid structures [17,24,122,123]. Hydrophobicity is an important determinant of the cytotoxicity of bile acids [124]. As plant compounds show increased interaction for hydrophobic bile acids, bile acid interactions, e.g., by adsorption, may change the availability of cytotoxic bile acids in the colon [82].

## 7. Conclusions

A fundamental understanding of the mechanisms of the interaction between primary and secondary bile acids and plant compounds is needed to improve the overall understanding of how diet modulates bile acid metabolism. Two main mechanisms of interaction can be concluded from the current state of research. Bile acids and plant compounds are either associated or complexed at a molecular level or increased viscosity reduces micellar mobility of bile acids. For both interaction mechanisms, an increased affinity towards hydrophobic bile acids was revealed. On the one hand, the constant pattern observed for molecular interactions indicates a common underlying mechanism based on hydrophobic interactions. On the other hand, dependency of bile acid retention on bile acid hydrophobicity in viscous matrices may be linked to the micellar properties of bile acids.

Due to the similar influence of viscosity-related and molecular interactions on the reduction of bile acid reabsorption, the differentiation of these effects in in vivo studies is impaired. To close the gap between the interaction mechanisms summarised in this review and the observed physiological outcomes, collaborative research activities through transdisciplinary approaches are required. In vitro approaches mimicking the physiological environment in the small intestine as well as structural techniques offer potential to elucidate the mechanistic principals of interactions in more detail. To verify if in vitro results accurately reflect complex in vivo scenarios, targeted in vivo studies should be conducted based on, and accompanied by, in vitro assessments. Future research further needs to clarify the complex interplay between the interaction of plant compounds and bile acids, the microbial changes of bile acids, the fermentation of indigestible plant compounds, and the consequences on the gut microbiome–bile acid axis.

To elucidate mechanisms behind physiological effects, many studies focused on isolated plant compounds, such as dietary fibres. However, investigations of isolated dietary fibre structures often conflict with results achieved for more complex fibre-enriched ingredients or food matrices. This may be caused by contributions from other plant compounds. While it is unclear whether plant proteins contribute to interactions with bile acids, there is growing evidence that phytochemicals, especially polyphenols, may contribute to bile acid sequestering effects of plant-based ingredients and foods. Polyphenols are known to be associated with plant proteins and dietary fibres. Further research thus needs to address combined mechanisms of interactions between bile acids and these plant compounds incorporated in intact food matrices, especially focusing on the influence of digestion on the stability and bioaccessibility of polyphenols. Furthermore, consequences resulting from mechanical, thermal, and chemical treatments need to be considered to enable the development of strategies for improved plant food processing.

## Figures and Tables

**Table 1:** Critical micelle concentration (CMC), aggregation number (N_agg_), and hydrophobicity of main conjugated bile acids abundant in the human bile acid pool.

Bile Acid	CMC (mM) ^1^	N_agg_^1^	Hydrophobicity ^2^
Glycocholic acid (GCA)	4	9	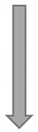
Taurocholic acid (TCA)	3–18	3–7	
Glycochenodesoxycholic acid (GCDCA)	1–2	15	
Taurochenodesoxycholic acid (TCDCA)	0.9–7	5–26	
Glycodesoxycholic acid (GDCA)	1–2	13–16	
Taurodesoxycholic acid (TDCA)	2–3	12–19	

^1^ As summarised by Parker et al. [20], taken from Madenci and Egelhaaf [33]; ^2^ taken from Heuman [34].

**Table 2:** In vivo and in vitro analysis of interactions between bile acids and plant compounds: approaches, benefits, and limitations.

Approach to Study Bile Acid Interactions	Details	Benefits	Limitations	Reference
Human studies	Bile acid analysis of faecal samples	Holistic assessment of effects on primary and secondary bile acid compositions in the colon	Transformation and reabsorption of bile acids in the colon Compensatory physiological processes	[2,31]
Human ileostomy studies	Bile acid analysis of ileal contents	Shorter and less variable transit time Minimal bacterial degradation of plant compounds and bile acids Short-term studies on bile acid metabolism	Availability of human ileostomy subjects Transferability of long-term effects on physiological processes in subjects without ileostomy	[12,26]
Animal models	Bile acid analysis of contents of intestinal sites or faecal samples	Understanding of bile acid concentrations along the intestinal tract Holistic assessment of effects on primary and secondary bile acid compositions	Deviating bile acid profiles in animals Compensatory physiological processes Transferability to human physiological processes	[6,43]
In vitro models based on centrifugation	Bile acid analysis in supernatant	Easily applicable High prevalence in literature	Variations regarding the use and parameters of in vitro digestion (i.e., critical micelle concentrations of bile acids not considered) Coverage of viscosity-related effects Applicability for soluble plant compounds	[8,14]
In vitro models based on dialysis	Bile acid transport across a dialysis membrane	Differentiation of viscosity-related and molecular bile acid interactions Applicability for soluble plant compounds Bile acid concentrations above critical micelle concentrations	Simplified model of unstirred water layer Estimation of physiological concentrations and viscosity	[10,47]
Structural in vitro techniques	Nuclear magnetic resonance, microcalorimetry, etc.	Assessment of molecular interactions Elucidation of molecular mechanisms	Transferability to physiological processes Coverage of viscosity-related effects	[5,48]

## Abbreviations

CA	cholic acid
CDCA	chenodesoxycholic acid
CMC	critical micelle concentration
CYP7A1	cholesterol 7α-hydroxylase
DCA	desoxycholic acid
EGCG	(−)-epigallocatechin-3-gallate
FXR	farnesoid X receptor
GLP-1	glucagon-like peptide-1
LCA	lithocholic acid
NMR	nuclear magnetic resonance
